# Electromagnetic Wave-Absorbing and Bending Properties of Three-Dimensional Gradient Woven Composites with Triangular Sections

**DOI:** 10.3390/polym14091745

**Published:** 2022-04-25

**Authors:** Huawei Zhang, Xinghai Zhou, Yuan Gao, Ying Wang, Yongping Liao, Liwei Wu, Lihua Lyu

**Affiliations:** 1 School of Textile and Material Engineering, Dalian Polytechnic University, Dalian 116034, China; zhanghuawei199411@163.com (H.Z.); zhouxh@dlpu.edu.cn (X.Z.); gaoyuan@dlpu.edu.cn (Y.G.); wy104020@163.com (Y.W.); liaoyp@dlpu.edu.cn (Y.L.); 2 Ministry of Education Key Laboratory for Advanced Textile Composite Materials, Tiangong University, Tianjin 300387, China

**Keywords:** 3D honeycomb gradient woven fabric, CB/CIP, carbon fibers, mechanical property, EM wave-absorbing property

## Abstract

In order to solve defects such as poor integrity, delamination failure, and narrow absorption bandwidth, three-dimensional (3D) gradient honeycomb woven composites (GHWCs) with triangular sections were designed and prepared. Three-dimensional gradient honeycomb woven fabric was crafted with carbon fiber (CF) filaments and basalt fiber (BF) filaments as raw materials on an ordinary loom. Then, the 3D honeycomb woven fabric filled with rigid polyurethane foam was used as the reinforcement, and epoxy resin (EP) doped with carbon black (CB) and carbonyl iron powder (CIP) was conducted as the matrix. The 3D GHWC with triangular sections, which had both EM-absorbing and load-bearing functions, was prepared by the VARTM process. Through the macro test and micro characterization of 3D GHWCs with triangular sections, the overall absorbing properties and mechanical properties of the materials were analyzed. Moreover, the EM-absorbing mechanism and failure mode of the materials were clarified in this work. The results indicated that the CF filament reflective layer effectively improved the EM-absorbing and mechanical properties. Adding a CB/CIP-absorbing agent enhanced the overall EM-absorbing property but reduced the mechanical properties. The increasing number of gradient layers increased the maximum bending load, but the EM-absorbing performance first increased and then decreased. When the thickness was 15 mm, the maximum bending load was 3530 N, and the minimum reflection loss (RL_min_) was −21.6 dB. The synergistic effects of EM-absorbing and mechanical properties were the best right now. In addition, this work provided a feasible strategy that adjusting the type of absorber and gradient aperture size ratio could meet the unique requirements of absorbing frequency and intensity, which has excellent application prospects in civil and military fields.

## 1. Introduction

In the era of the interconnection of all things, wireless technology has entered all aspects of life. The popularization of smart electronic products, intelligent textiles, and 5G communication are excellent carriers of electromagnetic wave propagation [1]. They have brought great convenience to people’s production and life, but they also have a huge potential threat [2]. EM interference has been causing significant damage to people’s electronic equipment and health [3]. Therefore, developing efficient and multifunctional EM-absorbing materials has become an urgent need. The traditional absorbing materials studied in the past have effective absorbing properties; however, they also have the defects of large thickness, high density, low EM-absorbing intensity, narrow absorbing bandwidth, and poor mechanical bearing capacity [4]. Aiming at these defects, researchers have proposed gradient EM-absorbing composites with a multidimensional structure. A pyramid structure [5], honeycomb structure [6], and multilayer stepped structure [7] were used in multidimensional gradient EM-absorbing composites for reducing the thickness and density. Duan et al. [8] prepared a pyramid structure gradient EM-absorbing composite with flake carbonyl iron particles and polyether ether ketone as raw materials by fused deposition modeling of 3D printing technology. The experimental and simulation results showed that when the thickness was 10 mm, the effective absorption bandwidth was as high as 5.1–40 GHz. Pei et al. [9] prepared a honeycomb structure gradient EM-absorbing composite by 3D printing technology. The absorbents were CIP and flaky Cu, and the matrix was polyethylene terephthalate glycol. The simulation and experimental results showed that the reflection loss was less than −10 dB from 8 to 12 GHz, and the RL_min_ was −28 dB. Luo et al. [10] prepared a gradient EM-absorbing composite with multilayer stepped structures by 3D printing technology and impregnation methods. The EM-absorbing properties of composites with different gradient structures were studied using experiments and simulation. The experimental results showed that the composites had excellent EM-absorbing properties at 1–18 GHz, and the EAB was as high as 14.06 GHz. In the above research, the manufacturing process of gradient structure EM-absorbing composites adopted 3D printing technology [11] and the impregnation lamination process [12]. There was a fatal mechanical defect in both processes. When the material was under an alternating external force or a high temperature and humidity environment, delamination failure would occur, which was offensive to the purpose that structural EM-absorbing composites needed to integrate the bearing function and EM-absorbing function. Therefore, the above experimental studies showed that the multidimensional structure enhanced the EM-absorbing properties of composites effectively; however, for the mechanical defects of microwave-absorbing composites, no clear solution has been given in past research.

Hence, the 3D GHWC with triangular sections was developed in this work. Taking the 3D gradient honeycomb woven fabric as the reinforcement could effectively improve the integrity of the composites and avoid delamination failure. Three-dimensional gradient honeycomb woven fabric led to the vertical yarn system in the weaving process, which connected the upper and lower fabrics to form a whole and improve the mechanical properties of the composites. Many research findings have demonstrated that 3D woven fabric could enhance the integrity of composites and solve the defect of delamination failure [13,14]. Because of the excellent mechanical properties of woven composites, woven composites have been widely used in industry [15,16]. Lyu et al. [17] compounded 3D honeycomb woven fabric with EP to prepare 3D honeycomb woven EM-absorbing composites. The CIP/CB EM-absorbing agents were added to EP. The experimental results showed that the material had good overall properties without delamination. Zhu et al. [18] prepared 3D woven tubular composites by compounding 3D tubular woven fabrics with epoxy resin. Through finite element simulation and an axial compression test, the mechanical properties and failure modes of the materials were analyzed. The results showed that the maximum load of 3D woven tubular composite with a 2 mm thickness was 10,578 N, which proved that the 3D tubular woven fabric had a significant influence on the mechanical properties. Bayraktar et al. [19] manufactured a 3D honeycomb woven composite and conducted a low-speed impact test on specimens. The results showed that the absorbing energy of the honeycomb structure was more visible than that of the plate specimen. The past research mainly focused on the mechanical properties of 3D woven composites. For the research on electromagnetic properties, 3D woven composites with a uniform aperture were studied, but the 3D woven composites with a gradient aperture structure have not been studied until now. Therefore, it was vital to study the mechanical and EM-absorbing properties of 3D GHWCs.

Therefore, 3D gradient honeycomb woven fabrics with different structural parameters were designed and woven on ordinary looms in this work. The 3D gradient honeycomb woven fabrics were compounded with EP doped with CIP and CB by the VARTM process, and then 3D GHWCs with triangular sections were prepared. The 3D GHWC with triangular sections had three significant advantages. Firstly, taking 3D gradient woven fabric as a reinforcement solved the defect of delamination failure in gradient EM-absorbing composites. Secondly, the absorbing frequency and absorbing intensity can be controlled to meet different practical needs by adjusting the 3D gradient honeycomb woven fabric’s structure and/or the type of EM-absorbing agent. Thirdly, the 3D GHWC with triangular sections had the advantage of integrating bearing and EM-absorbing functions to meet broad application prospects. The 3D GHWC had the characteristic of integrating bearing and EM absorption, which meant it could be widely used in anti-electromagnetic pollution, and anti-electromagnetic interference environments such as aerospace, ship transportation, etc. Meanwhile, its characteristics of mechanical structure and frequency selection have unique advantages in the construction of radar masks and EM anechoic chambers.

## 2. Experiments

### 2.1. Material Design

**Figure 1:**
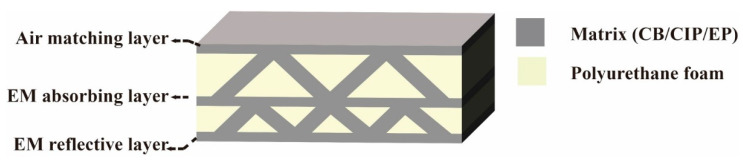
The schematic diagram of the structural design.

The structure of the 3D GHWC with triangular sections was designed into an air matching layer, an EM-absorbing layer, and an EM-reflective layer. The schematic diagram of the structural design is shown in Figure 1. The EM-absorbing performance of 3D GHWCs came from the impedance matching between the air matching layer and air, the effective EM-absorbing capacity of the EM-absorbing layer, and the EM-reflective performance resulting from the conductive network in the EM-reflective layer.

### 2.2. Materials and Instruments

CIP powders (the particle diameter ranging from 1 to 3 μm) were from Nangong Xindun Alloy Welding Material Spraying Co., Ltd., Nangong, China, and CB powders originated from Suzhou Dinero Technology Co., Ltd., Suzhou, China, with a diameter of 20 nm. The 800 tex BF filament was provided by Zhejiang Shijin Basalt Fiber Co., Ltd., Hangzhou, China. The 800 tex carbon fiber (CF) filament (SYT49) was produced by Zhongfu Shenyang Carbon Fiber Co., Ltd., Lianyungang City, China. EP (692K-A) and the curing agent (692K-B) were supplied by Shenzhen No. 1 Advanced Materials Co., Ltd., Shenzhen, China. All materials and reagents needed no further purification.

The field emission scanning electron microscopy (SEM, Model JSM-7800F, JEOL, Tokyo, Japan) and transmission electron microscopy (TEM, Model JEM-2100UHR, JEOL, Tokyo, Japan) was adopted to describe the morphologies and microstructures of the CIP and CB, respectively. Moreover, SEM was used to characterize the dispersion of the CB/CIP in EP. According to the standard GB/T 9341-2008, the bending properties of the 3D GHWC with triangular sections were measured with the test speed of 2 mm/min by the universal testing machine (Model TH-8102S, TOPHUNG, Suzhou, China). Three samples of each type were taken for testing at ordinary temperature, and the final results of the experiment were averaged.

According to the standard GJB 2038-1994, the electromagnetic parameters of all structural layers were tested from 2 to 18 GHz at room temperature through an Agilent vector network analyzer (8720B) with the coaxial transmission line method. The sample was cut into a tube that had a 3.04-mm inner diameter, 7.00-mm outer diameter, and 2.50-mm height by a carving machine. The Agilent vector network analyzer (Model E5071c) was linked to two standard gain horn antennae that generated microwaves from 1 to 18 GHz. According to these, the RL property of the 3D GHWC with triangular sections was tested. The sample size was 180 × 180 mm.

### 2.3. Material Preparation

**Figure 2:**
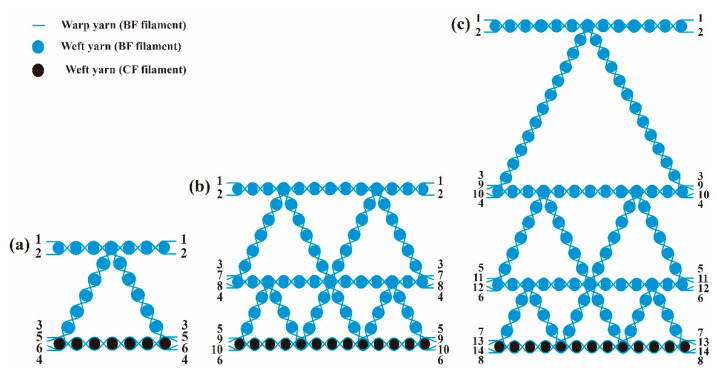
(**a**) Warp structural drawing of one-layer fabric; (**b**) warp structural drawing of two-layer fabric; (**c**) warp structural drawing of three-layer fabric.

Three-dimensional gradient honeycomb woven fabric was designed by warp structural drawings according to actual needs. The warp section of the fabrics was designed as triangles, and Figure 2 shows the warp structural drawings of 3D gradient honeycomb woven fabrics with different structures.

According to the design of the absorbing structure, except that the bottom weft yarn adopted the 800 tex CF filament, the rest of the yarn used the 800 tex BF filament. To obtain the best mechanical properties, the basic organization was plain weave, and the binding organization adopted rib weave. According to the warp structural drawings, the parameters of 3D gradient honeycomb woven fabrics with different layers were calculated. Table 1 indicates the specific operating parameters.

**Figure 3:**
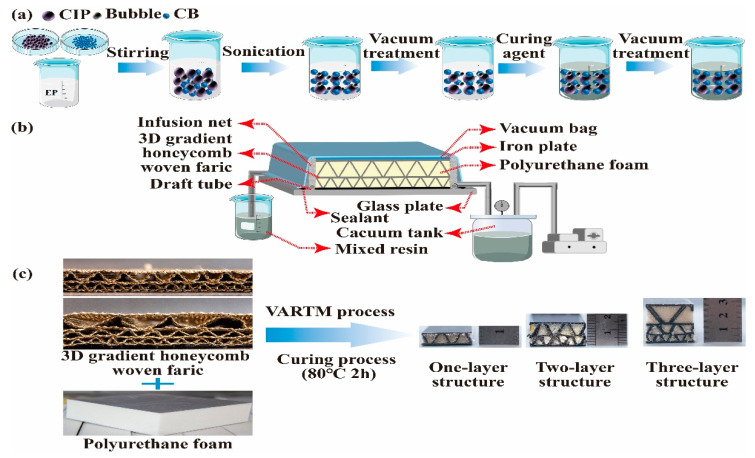
(**a**) Illustration of the microwave-absorbing resin preparation; (**b**) schematic of VARTM process; (**c**) the fabrication procedure and the physical maps of 3D GHWCs with triangular sections.

In Figure 3, the 3D GHWCs with triangular sections were prepared by the VARTM process. In Step 1, as Figure 3a shows, the CB, CIP, and EP with a mass percentage of 1%:45%:54% were mixed and stirred mechanically for 9 min in a beaker. In Step 2, the mixture was ultrasonically dispersed for 25 min and then evacuated for 25 min in a vacuum oven (24 °C) to cool and defoam. In Step 3, the curing agent was added to the mixed liquor with a mass percentage (EP: curing agent = 25%:75%), and then stirred mechanically for 7 min. In Step 4, it was evacuated for 1 h in a vacuum oven (24 °C). As Figure 3b shows, the 3D gradient honeycomb woven fabric (200 × 200 mm) filled with polyurethane foam was placed onto the glass plate for an air-tight test. The air-tight test was sustained under a vacuum environment for 4 h. The obtained mixed resin liquor was infused into the vacuum area until several consecutive resins were discovered in the draft tube. Finally, as Figure 3c shows, the glass plate was put into the air-circulating oven at 80 °C for 2 h. Table 2 displays the physical parameters of 3D GHWCs with triangular sections.

## 3. Results and Discussion

### 3.1. Dispersion of Absorbing Agent in Epoxy Resin (EP)

**Figure 4:**
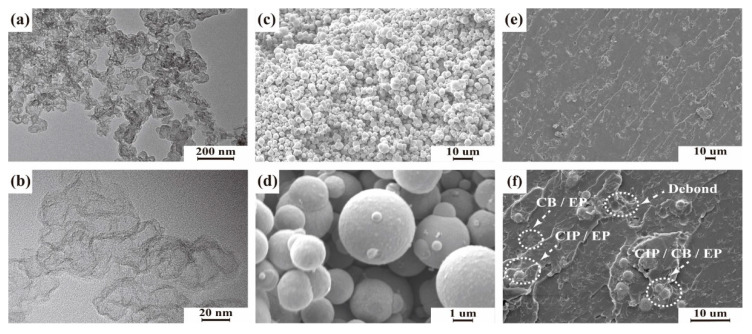
Morphologies of the composites. (**a**,**b**) TEM graphics of the CB powders; (**c**,**d**) SEM images of the CIP powders; (**e**,**f**) SEM images of the fracture surface of the CB/CIP/EP.

The uniform dispersion of the CIP and CB in EP was significant to the mechanical and EM properties of the 3D GHWC with triangular sections. The bonding interface between particles and EP directly affected the mechanical properties. The conductive network structure formed by CIP and CB would affect the dielectric properties. Meanwhile, the agglomeration effect of particles changed the size and shape of particles and then affected the mechanism of magnetic loss. Figure 4 shows the micromorphology of CB, CIP, and CB/CIP/EP.

To observe nano CB more clearly, TEM was used to characterize the micromorphology of CB. As shown in Figure 4a,b, the shape of CB was a flake structure, the size was around 20–30 nm, and the overall distribution tended to stack with each other, which was conducive to the formation of a conductive network in the later stage. Micron CIP and CB/CIP/EP were characterized by SEM.

As shown in Figure 4c,d, the shape of CIP was approximately circular, and the particle size was between 3 and 5 μm. The smooth outer surface had the advantage of distribution and crosslinking with EP. According to the SEM diagram of CB/CIP/EP in Figure 4e,f, the dispersion of CIP was relatively uniform, while the size and shape of CB had changed dramatically due to the nano agglomeration effect. The particle size of CB after agglomeration was 10–15 μm. However, some evenly dispersed CB with non-agglomeration was undetected in the EP due to its small size. In addition, some CIP fell off the surface of CB/CIP/EP, indicating that the interfacial adhesion between CIP and EP needed to be improved.

### 3.2. EM-Absorbing Properties of 3D GHWCs with Triangular Sections

#### 3.2.1. Influence of Structural Layer on EM-Absorbing Performance

**Figure 5:**
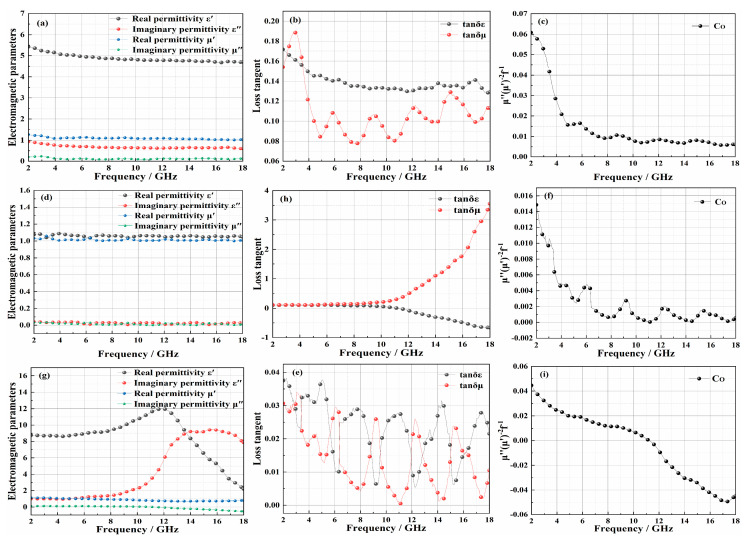
(**a**–**c**) EM parameters of matching layer and EM-absorbing layer; (**d**–**f**) EM parameters of rigid polyurethane foam; (**g**–**i**) EM parameters of the reflective layer.

To further study the influence of structure layers on EM-absorbing performance and clarify the EM-absorbing mechanism, the EM parameters of the matching layer, EM-absorbing layer, rigid polyurethane foam, and the reflective layer are shown in Figure 5. The real part of electromagnetic parameters (ε′, μ′) represents the energy storage capacity of materials for electromagnetic waves, and the imaginary part (ε″, μ″) represents the dissipation capacity of electromagnetic waves [20].

The EM-absorbing properties of materials were composed of electrical loss and magnetic loss. The magnetic loss tangent (tanδε = ε″/ε′) described the magnetic loss performance of the material, and the electrical loss tangent (tanδμ = μ″/μ′) represented the electrical loss performance of the material [20]. According to BF and EP being excellent wave-transparent materials, the EM-absorption properties of the EM-absorbing layer and air matching layer mainly came from CIP and CB. As shown in Figure 5a,b, the electric loss of the materials was more significant. The electric loss comprises dielectric loss, interfacial polarization, and polarized relaxation [21]. The CB and CIP formed a conductive bridge, which provided a path for the movement of free charge. According to this, the dielectric loss and polarized relaxation of the material increased. At the same time, the interfacial properties between CB, CIP, EP, and BF increased the interfacial polarization. With the increase of frequency, the value of ε′ decreased gradually, which showed that the material had a slight frequency-dependent dielectric effect and dispersion effect. Still, the reduced ε′ was also conducive to the impedance matching performance and met the design requirements of the air matching layer [22]. Magnetic loss consists of eddy current loss, natural resonance, hysteresis loss, and domain wall resonance [23]. Since the test frequency band was 2–18 GHz, the domain wall resonance effect cannot occur, and the hysteresis loss is negligible. At the same time, when the eddy current loss effect is the only factor of magnetic loss, the value of Co(μ″(μ′)^−2^f^−1^) is a fixed value independent of frequency [24]. As indicated in Figure 5c, the value of Co decreased with the frequency. Therefore, the EM-absorbing properties of the air matching layer and the EM-absorbing layer mainly came from dielectric loss, interface polarization and polarization relaxation, eddy current loss, and natural resonance. From the EM parameters of polyurethane foam in Figure 5d,e, the polyurethane foam had almost no EM absorption. It was excellent wave-transparent material and could be used as a structural support. Figure 5g–i shows the EM parameters of the reflective layer. The weft yarn of the reflective layer adopted the CF filament, which could form an efficient conductive network between CB and CIP. Moreover, the interfacial properties between CB, CIP, EP, CF filament, and BF filament enhanced the interfacial polarization. Therefore, the reflective layer had a very high dielectric constant. In Figure 5h, the value of tanδε was negative and the value of tanδμ was positive. Therefore, the reflective layer acted on the EMW in the form of reflection, which met the design requirements. Meanwhile, in Figure 5f,i, the values of Co all decreased with the frequency. Therefore, the polyurethane foam and the reflective layer had the same magnetic loss with the EM-absorbing layer. In conclusion, according to the EM parameters of the matching layer, EM-absorbing layer, and reflective layer, the prepared 3D GHWC with triangular sections met the structural design requirements.

#### 3.2.2. Reflection Loss

**Figure 6:**
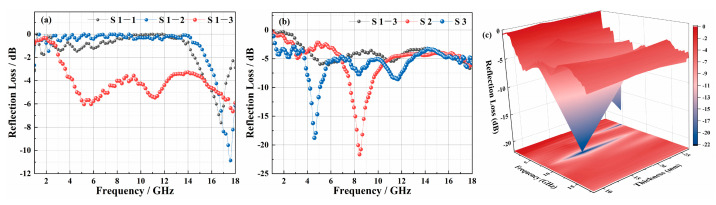
(**a**) The reflection loss of S1-1, S1-2, and S1-3; (**b**,**c**) the reflection loss of the different-layer 3D GHWCs with triangular sections.

According to different structural parameters, five samples (S1-1, S1-2, S1-3, S2, and S3) were designed. To further study the effects of the reflective layer, EM-absorbing agent, and the number of structural layers on the EM-absorbing properties of 3D GHWCs with triangular sections, the reflection loss of five samples was tested (Figure 6).

The RL_min_ and EAB were essential indexes to evaluate the EM-absorbing performance of materials. In Figure 6a, because the reflective layer had the dual functions of absorption and reflection, the RL_min_ decreased from −7 dB (S1-1) to −11 dB (S1-2) with the addition of a reflective layer. The overall EM-absorbing performance of the material had been greatly improved with the addition of an EM-absorbing agent. Still, the absorption peak value had decreased due to the impedance matching performance of the surface layer as it decreased. At this time, the reflection and refraction of the EM wave occurred, and the EM wave entering the material decreased. Furthermore, the number of structural layers was the critical factor that affected the EM-absorption performance of 3D GHWCs. In Figure 6b, the RL_min_ of the one-layer structural S1-3 was −6.6 dB (5.1 GHz), and the absorption bandwidth < −5 dB was 3.1 GHz (4.6–6.7 GHz, 10.5–11.5 GHz). The RL_min_ of the two-layer structural S2 was −21.6 dB (8.4 GHz), the EAB was 1.8 GHz (7.6–9.4 GHz), and the absorption bandwidth below −5 dB was 4.1 GHz (11.0–6.9 GHz). The RL_min_ of the three-layer structural S3 was −18.9 dB (4.6 GHz), the EAB was 0.9 GHz (4.2–5.1 GHz), and the absorption bandwidth below −5 dB was 9.4 GHz (3.7–6.7 GHz, 6.9–12.9 GHz, 16.7–17.1 GHz).

**Figure 7:**
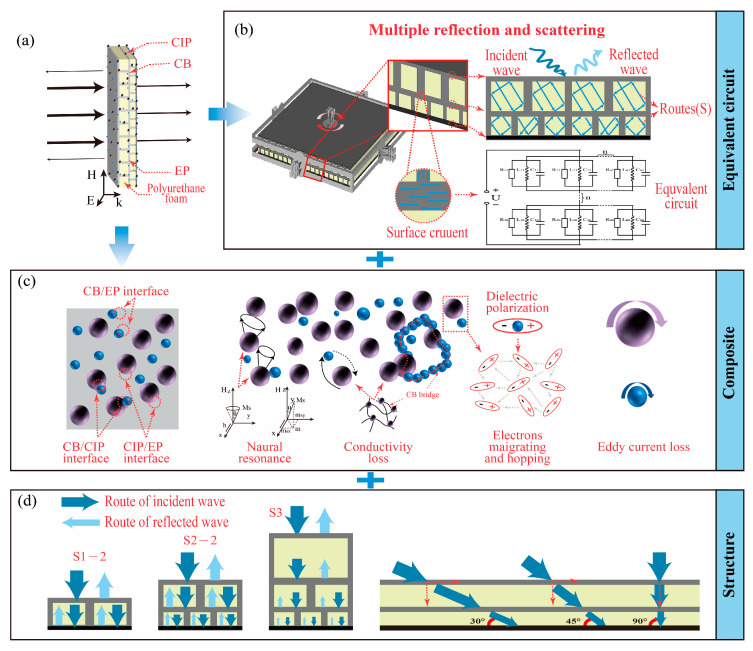
(**a**) The schematic diagram of EM wave incidence; (**b)** the schematic diagram of the equivalent circuit; (**c**) the micro schematic diagram of EM-absorbing mechanisms; (**d**) the structural schematic diagram of EM-absorbing mechanisms.

Through the comparative analysis of the experimental results between S1-3, S2, and S3, when the material structure was raised from one layer to two layers, the RL_min_ and EAB had significantly improved due to the gradient structure, which increased the impedance matching performance and the reflection loss path. Because the smaller aperture structure could enhance the EM-absorbing performance, the absorbing behavior of the bottom-layer structure had a significant impact on the whole EM-absorbing performance. As is shown in Figure 7d, when the number of structural layers increased from two to three, the multilayer structure led to more reflection behavior. The EMW entering the bottom layer decreased sharply, so the RL_min_ decreased. At the same time, the quarter-wavelength theory [25], that the increased material thickness makes the absorption peak move to low frequency, resulted in the reduction of the EAB. However, the absorption bandwidth < −5 dB was significantly improved, which proved that the gradient structure effectively improved the impedance matching performance. In summary, S2 had the best EM-absorbing performance among all samples. Meanwhile, S1-3 and S3 had similar trends to the EM-absorbing curves of S2. To more clearly study the influence of thickness on the EM-absorbing performance of the 3D GHWC, the 3D model diagram was drawn in Figure 6c. With the continuous increase of thickness, the RL_min_ first decreased and then increased, and the EM-absorbing peak moved to low frequency. This result was consistent with Figure 6b. Finally, comparing the RL_min_ and EAB values of previous research results, it could be seen that the EM-absorbing performance of 3D GHWCs met the conventional requirements of the application. At the same time, 3D GHWCs had a certain advantage for the specific EAB design [26].

#### 3.2.3. EM-Absorbing Mechanisms

Figure 7 is the schematic diagram of EM-absorbing mechanisms. According to the above conclusions and analysis, the possible EM-absorbing mechanism of 3D GHWCs with triangular sections was summarized. As is shown in Figure 7, the EM-absorbing mechanism of composite materials mainly came from reflection path loss, electrical loss, and magnetic loss.

Figure 7a is a schematic diagram of EMW incidence. The EMW was perpendicular to the upper surface of the 3D GHWC. In Figure 7b, the equivalent circuit formed by the surface currents of different layers produced multiple reflection and scattering phenomena. Therefore, the reflection paths of different structural layers were different. The multiple reflection and scattering behavior, which stemmed from the reflection path loss, existed not only between the upper and lower structural layers but also between the EM-absorbing particles. Meanwhile, in Figure 7c, the dielectric loss and polarized relaxation in electrical loss came from the conductive network formed between CB, CIP, and CF filaments. Five different media (CB, CIP, BF filament, CF filament, and EP) also enhanced the interfacial polarization effect. Moreover, the natural resonance effect and eddy current loss constituted the magnetic loss. In Figure 7d, the most critical reflection path loss originated in the structural design. When the number of structural layers increased, the multilayer structure led to more reflection behavior.

In general, the EM-absorbing mechanism of the 3D GHWC with triangular sections benefited from the synergy of multiple media and structural design.

### 3.3. Bending Properties of 3D GHWCs with Triangular Sections

#### 3.3.1. Load–Displacement Curves

**Figure 8:**
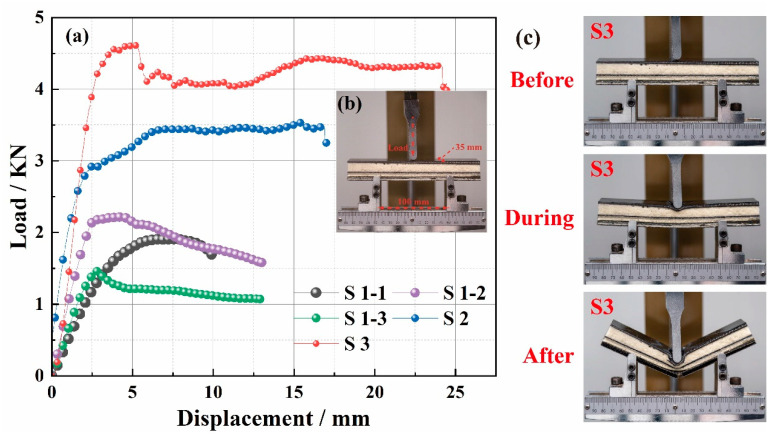
The bending behaviors of the 3D GHWC with triangular sections. (**a**) Bending load–displacement curve; (**b**) experiment settings; (**c**) comparing images of before, during, and after bending.

Figure 8 shows the three-point bending experimental process and results. According to Figure 8a, the maximum bending loads of the five samples were S1-1 = 1910 N, S1-2 = 2215 N, S1-3 = 1465 N, S2 = 3530 N, and S3 = 4616 N, respectively. First, compared with S1-2 and S1-1, the addition of the CF filament enhanced the maximum bending load of S1-2, which was due to the higher modulus and strength of the CF filament. Next, compared with S1-2 and S1-3, the maximum bending load of S1-2 decreased with the addition of an absorbing agent, which was because the absorbing agent destroyed the interfacial properties between resin and fiber. Moreover, compared with S1-3, S2, and S3, the maximum bending load of the material increased with the increasing number of structural layers. This was because the increased 3D gradient woven fabric structure improved the overall mechanical properties of the 3D GHWC when the number of structural layers increased. Figure 8b is the schematic diagram of parameter settings of the three-point bending test. The width of the sample was 35 mm, and the span was 100 mm. Meanwhile, the test was carried out in the form of upper indenter loading. In addition, all samples showed a yield peak in Figure 8a, and each sample broke during the bending process in Figure 8c.

#### 3.3.2. Failure Modes

**Figure 9:**
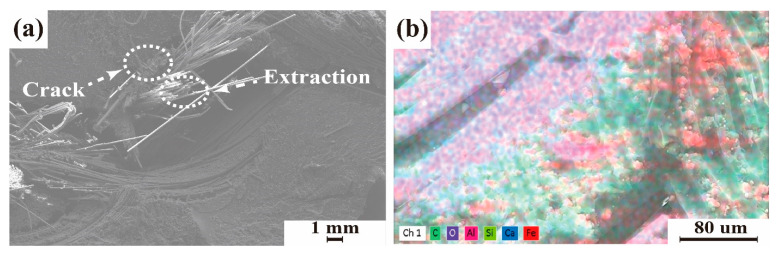
SEM of the fracture surface. (**a**) Microscopic morphology of fracture surface; (**b**) energy spectrum of the fracture surface.

Figure 9 shows the micromorphology and energy spectrum of the fracture surface of 3D GHWCs with triangular sections. From the micromorphology in Figure 9a, the interior of the yarn was not completely soaked during the pouring process of the composite. Therefore, the fibers around the yarn were broken and damaged, and the fibers in the middle of the yarn were pulled out. In Figure 9b, the energy spectrum showed that the absorbing agent was well dispersed in the matrix, but there was still an agglomeration effect. The fibers were pulled out and had slipped due to the poor bonding performance between the resin and fiber in the agglomeration area.

**Figure 10:**
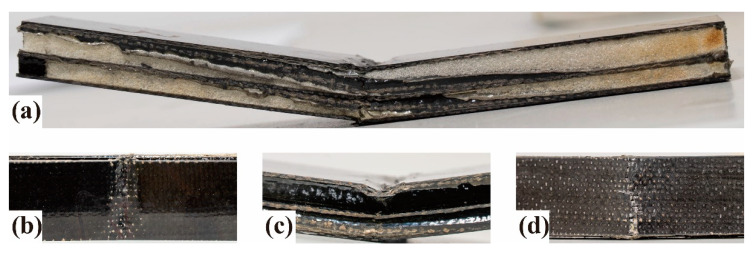
Fracture morphology of the composites. (**a**) Overall view of S2; (**b**) partial top view of S2; (**c**) partial side view of S2; (**d**) partial bottom view of S2.

Combined with the load–displacement curve of S2 in Figure 8a and the physical diagram of the fracture surface of S2 in Figure 10a, the material showed an elastic-plastic deformation state in the whole process. Meanwhile, the upper layer was a compressive failure in Figure 10b, the middle layer was a shear failure in Figure 10c, and the bottom layer was a tensile failure in Figure 10d.

The bending test could be divided into four stages. In the initial loading stage, the composite bore the bending load as a whole in the initial displacement, and a linear bending load-displacement curve appeared. The composite had no obvious fracture, but microcracks appeared in the stressed part due to the different shape variables between the fiber and resin. Therefore, the composites had good linear elastic properties. In the second stage, as the upper indenter declined, the compressive shrinkage force affected the upper-layer material, the sheer force affected the middle-layer material, and the tensile force affected the bottom-layer material. Due to the different pore structures of the upper and bottom layers, the bottom structure with a smaller pore size could bear a more significant load due to its densification structure and the addition of CF filaments. Therefore, the structure with a large pore diameter took the lead in compression failure, and the slope of the curve decreased. Because the bottom was not damaged, the bending load–displacement curve of the material was still in a linear state. In the third stage, as the upper indenter declined, the microcracks in the upper layer transferred to the bottom layer, which damaged the interfacial bonding performance between resin and fiber. The BF filaments in the middle layer began to break and get pulled out; nevertheless, the CF filaments in the bottom layer were not damaged. The load–displacement curve reached the maximum value, and the stress was stable at the peak value. In the last stage, as the upper indenter continued to decline, the tensile failure of the bottom carbon fiber appeared, a large number of CF filaments broke and were pulled out, the whole material broke, and the load dropped rapidly. According to the physical diagram of the fracture surface in Figure 10, and compared with the traditional gradient structure EM-absorbing composites studied in the past, there was no obvious delamination after the 3D GHWC was damaged, which proved that the integrity of the material was good [27].

## 4. Conclusions

In order to prepare the composite material with the integration of loading and EM absorbing, the 3D GHWCs with triangular sections were prepared with CB/CIP/EP/BF/CF. In the VARTM process, adjusting the type of EM absorber and the aperture ratio of the gradient structure can effectively control the EM-absorbing frequency band and EM-absorbing intensity. Furthermore, the work experimentally studied the effects of the reflective layer, EM absorber, and the number of structural layers on the mechanical and EM-absorbing properties of 3D GHWCs with triangular sections.

In terms of EM-absorbing performance, the reflection effect of the reflective layer reduced the RL_min_, and the overall EM-absorbing performance was significantly improved by adding an EM-absorbing agent. Meanwhile, the increase of structural layers strengthened the impedance matching performance and the reflection behavior, and so the EM-absorbing performance was improved first and then reduced. In addition, the EM-absorbing mechanism and mechanical failure mode of the material were also deeply analyzed. The EM-absorbing mechanism of 3D GHWCs with triangular sections came from electrical loss, magnetic loss, multiple reflections, and scattering effects.

In terms of mechanical properties, the addition of CF filaments promoted the maximum bending load of the materials. However, the addition of the EM-absorbing agent destroyed the interface between the resin and fiber and reduced the maximum bending load. Moreover, the increase of structural layers improved the maximum bending load. The mechanical failure mode of 3D GHWCs with triangular sections was mainly typical shear failure, which consisted of matrix cracking and fiber bundle fracture and extraction.

Finally, comparing the EM-absorbing performances and mechanical properties of all 3D GHWCs, the EM-absorbing and mechanical properties of S2 had the best synergy. The maximum bending load was 3530 N, the RL_min_ was −21.6 dB, and the EAB was 1.8 GHz.

## Figures and Tables

**Table 1:** Weaving parameters of 3D gradient honeycomb woven fabrics.

Thickness/mm	Linear Density/Tex	Layer Number of Fabrics	Weaving Density (yarn/10 cm)	
	Warp/Weft Yarns		Warp Density	Warp Density
8.5	800	1	150	180
15		2	250	308
26.5		3	350	450

**Table 2:** Physical parameters of 3D GHWCs with triangular sections.

Specimen	Thickness/mm	Number of Layers	EM-Reflective Layer	CB/CIP EM Absorbing Agent
S1-1	8.5	1	No	No
S1-2	8.5	1	Yes	No
S1-3	8.5	1	Yes	Yes
S2	15	2	Yes	Yes
S3	26.5	3	Yes	Yes
